# Risk of death or hospital admission among community-dwelling older adults living with dementia in Australia

**DOI:** 10.1186/1471-2318-14-71

**Published:** 2014-06-10

**Authors:** Emily (Chuanmei) You, David Robert Dunt, Vanessa White, Stephen Vander Hoorn, Colleen Doyle

**Affiliations:** 1 Centre for Health Policy, Melbourne School of Population and Global Health, The University of Melbourne, Victoria 3010, Australia; 2 The Wesley Research Institute, PO Box 499, Toowong Queensland 4066, Australia; 3 Centre for Advanced Imaging, The University of Queensland, Brisbane Queensland 4072, Australia; 4 Australian Institute for Primary Care & Ageing, Faculty of Health Sciences, La Trobe University, Victoria 3086, Australia; 5 Department of Mathematics and Statistics, Statistical Consulting Centre, The University of Melbourne, Victoria 3010, Australia; 6 National Ageing Research Institute, PO Box 2127, Victoria 3050, Australia; 7 Australian Catholic University & Catholic Homes, Victoria 3065, Australia

**Keywords:** Death or hospital admission, Community-dwelling, Risk factors, Dementia

## Abstract

**Background:**

Older people living with dementia prefer to stay at home to receive support. But they are at high risk of death and/or hospital admissions. This study primarily aimed to determine risk factors for time to death or hospital admission (combined) in a sample of community-dwelling older people living with dementia in Australia. As a secondary study purpose, risk factors for time to death were also examined.

**Methods:**

This study used the data of a previous project which had been implemented during September 2007 and February 2009. The original project had recruited 354 eligible clients (aged 70 and over, and living with dementia) for Extended Aged Care At home Dementia program services during September 2007 and 2008. Client information and carer stress had been collected from their case managers through a baseline survey and three-monthly follow-up surveys (up to four in total). The principal data collection tools included Global Deterioration Scale, Modified Barthel Index, Instrumental-Dependency OARS, Adapted Cohen-Mansfield Agitation Inventory, as well as measures of clients’ socio-demographic characteristics, service use and diseases diagnoses. The sample of our study included 284 clients with at least one follow-up survey. The outcome variable was death or hospital admission, and death during six, nine and 16-month study periods. Stepwise backwards multivariate Cox proportional hazards analysis was employed, and Kaplan-Meier survival analysis using censored data was displayed.

**Results:**

Having previous hospital admissions was a consistent risk factor for time to death or hospital admission (six-month: HR = 3.12; nine-month: HR = 2.80; 16-month: HR = 2.93) and for time to death (six-month: HR = 2.27; 16-month: HR = 2.12) over time. Previously worse cognitive status was a consistent risk factor over time (six- and nine-month: HR = 0.58; 16-month: HR = 0.65), but no previous use of community care was only a short-term risk factor (six-month: HR = 0.42) for time to death or hospital admission.

**Conclusions:**

Previous hospital admissions and previously worse cognitive status are target intervention areas for reducing dementia clients’ risk of time to death or hospital admission, and/or death. Having previous use of community care as a short-term protective factor for dementia clients’ time to death or hospital admission is noteworthy.

## Background

Like other older people, people living with dementia prefer to stay out of hospital, live in their own homes and live life for as long as possible. Unfortunately, these frail older people are at high risk of hospital admissions, nursing home admissions, and death [1-3]. Hospital and nursing home admissions create considerable burden on health system resources, since they account for a large proportion of frail older adults’ social and health care expenditures, not to mention the considerable emotional cost for dementia carers or family members [2,4].

Therefore improving our knowledge of the risk factors associated with these adverse health events and implementing appropriate interventions can potentially reduce unnecessary use of expensive hospital and nursing home care, improve health outcomes of community-dwelling older people living with dementia, and finally save resources for our health system [2].

There have been many empirical and review studies examining the effects of risk factors on permanent nursing home admission [1,5-12]. In comparison, there have been fewer studies exploring risk factors for mortality of older people living in nursing homes or in the community [13-18].

With different sample size, target populations, follow-up periods, independent variables, measurement tools etc., these studies report that socio-demographic characteristics (such as age and gender), physical and cognitive functioning abilities, stress factors, chronic diseases, and other factors of clients and carers can be significant factors for those adverse health events. Among these significant factors, cognitive impairment, disabilities in activities of daily living (ADL) and instrumental activities of daily living (IADL), and dementia diagnosis are identified as relatively more consistent risk factors [9,10,19-23].

However, there have been few empirical studies exploring risk factors of hospital admissions or mortality especially among community-based frail older adults living with dementia. Population aging and older people preferring to live in the community lead to an increase in the number of dementia cases in the community, and associated increase in the demand of community-based dementia care. It is worthwhile to undertake research to determine risk factors of hospital admission and death among community-dwelling older people living with dementia, which may have important implications on community-based dementia care practice [24,25]. As described previously, reducing unnecessary hospital admissions and death among this specific population will benefit heath system resources, dementia care clients, and family members or carers.

It is important to examine the impact of risk factors on hospital admissions and death at different times since risk factors and/or their values, service arrangements and targeting interventions may change due to the change of frail older people’s functioning and health conditions over time [16,26].

In Australia, the Extended Aged Care at Home Dementia (EACHD) program/package is a case management program targeting community-dwelling frail older people with behavioural and psychological symptoms of dementia (BPSD). As an alternative to high residential care, this program aims to maintain clients at home safely for as long as possible, and specifically improve client outcomes (such as improving quality of life and avoiding death), reduce unnecessary use of intensive care (such as hospital care), and prevent permanent residential care placement [27].

Identifying risk factors of hospital admissions and death among EACHD clients and implementing interventions can have the following benefits, including [27-29]:

•Improving clients’ use of EACHD program services, functioning abilities and health outcomes

•Preventing EACHD clients’ inappropriate movements between hospital care and community aged care and therefore reducing burden on health system resources

•Reducing financial and emotional burden on individual families

Using a longitudinal data set generated by the “National Evaluation of the EACHD program” project, this study primarily aimed to determine risk factors (from socio-demographic, clinical and service use factors available from the data set) for time to death or hospital admission (a combined health event) in a sample of EACHD clients in the short term (six and nine months’ study periods) and longer term (16 months’ study period). Death or hospital admission was a parameter for client discharge from the EACHD program [26]. As the literature has explored risk factors of mortality, we examined risk factors for time to death as a secondary purpose of this study.

## Methods

This study used the data of the “National Evaluation of the EACHD program” project, which was a 16-month time-series study implemented between September 2007 and February 2009 [26]. All fieldwork methods were approved by La Trobe University Human Ethics Committee (07–084).

### Population of the original project and data collection

Participant recruitment was undertaken between September 2007 and 2008 and data collection continued until February 2009. According to the government guideline [30], participant eligibility criteria were:

•People aged 70 and over (or 50 and over if indigenous) living in the community or retirement villages.

•Having a higher level of residential care needs and the following characteristics: experiencing Behavioural and Psychological Symptoms of Dementia (BPSD); preferring to receive EACHD program services; and being able to live at home with the support and services provided by the program.

•Possibly having ADL and IADL disabilities and a higher level of care needs associated with their behaviours of concern, and facing the risk of unavoidable permanent nursing home admission.

However, it should be noted that at the initial stage of the EACHD program, not all 354 individuals enrolled by the original research project were 70 years old or over. Specifically, 27 clients were aged between 58 and 69, and 19 clients were aged between 58 and 64 (strictly speaking not “older people”). In addition, no indigenous people were enrolled even though they were included in the target population of the EACHD program.

Clients and carers’ information was collected from their case managers at baseline (the study entry), and every three months thereafter (up to four assessment points in total). Specifically, clients’ socio-demographic characteristics were collected at baseline. Clients’ medical diagnoses, physical and cognitive status, behavioural problems, services use, carers’ stress due to client BPSD etc. during the past three months were surveyed at baseline and each assessment point. A discharge survey was conducted if a client was discharged within the 16-month study period due to the following reasons: death, permanent nursing home admission, permanent nursing home admission and death, hospital admission, hospital admission and death, hospital admission and nursing home admission, and other unknown reasons.

The number of surveys per client ranged from one (only baseline survey) to five (baseline survey plus four follow-up surveys, or baseline survey plus three follow-up surveys and the discharge survey), depending on the enrolment time - later enrolees had fewer surveys.

Aiming to compile a national data set of at least 300 clients at the initial stage of the EACHD program, the original project stopped recruitment at 354 clients due to time constraints [26].

### Population of our study

We only used 284 participants’ data. The other 70 participants’ data were inappropriate for Cox regression analysis because 65 clients only had the baseline survey and five clients had baseline and discharge surveys occurring at the same dates. The 70 participants were lost to follow up according to the original project. Excluding these participants should not lead to obviously biased results because their baseline variables were similar to those of the 284 participants.

### Measures of our study

#### Outcome variables

Outcome variables included (inpatient) death or hospital admission (on either condition clients would be discharged) during six, nine, and 16 months after enrolment. Emergency department (ED) visits and outpatient hospital admissions were not included because clients experiencing these events remained using EACHD program services.

In our study, death and hospital admission were combined as one endpoint—death or hospital admission. One consideration was that time to hospital admission and time to death were likely to be dependent because poor health status potentially increases the risk of both hospital admission and death [31]. In addition in health and medical research, it is not uncommon for researchers to combine adverse health events, such as the permanent nursing home admission and death, and hospital admission and death [3,32-34]. Another consideration was that small numbers of clients experiencing hospital admission and death made it inappropriate to conduct analyses for the two outcomes separately (see details in Results section).

We also examined risk factors for time to death as among those clients experiencing death or hospital admission most were death cases. As described in the Background, we treated this as a secondary study purpose because there has been similar research on this topic. In addition, we were aware that the findings might be limited by inadequate sample size (see Sample size section below).

#### Time to death or hospital admission (and death)

##### Sixteen-month study period

Time to death or hospital admission was estimated as the number of days from the study entry (the date of completing the baseline survey) to the date of death or hospital admission. Clients who had not yet died or moved to hospital were censored at the date of the last survey (last follow-up survey, or discharge survey due to other reasons but not death or hospital admission).

##### Six and nine-month study periods

Time to death or hospital admission was counted as the number of days from the study entry to the date of death or hospital admission. Clients not experiencing death or hospital admission were censored at the date of the last survey if it occurred before the end of the study period. Or they were censored at the end of the study periods (at six months and nine months) if the last survey occurred after the end of the study periods.

#### Independent variables

Based on previous studies [6,14,18,20], and available information of the original project, our study included the following baseline variables for Cox regression analysis:

### Client variables

Socio-demographic characteristics: age, gender, birthplaces, income sources, first language, living arrangements, carer relationships, and carer status.

Use (0 = no; 1 = yes) of services: GP visits, ED visits, outpatient visits, inpatient hospital admissions, home nursing care, dementia specialist care, and community care services (including allied health, personal care, domestic care, information services, social support services and respite services).

Use of case management time: measured by number of hours.

Severity of medical conditions: According to the Charlson index of co-morbidity [35], different diseases of clients were weighted differently. For example, cerebrovascular diseases, diabetes, congestive heart failure etc. scored 1; renal impairment, tumours etc. scored 2; and metatastatic solid tumours scored 6. Based on the total scores of clients’ diseases, clients’ medical conditions were classified into no, mild (1 or 2 scores), moderate (3 or 4 scores) and severe conditions (over 4 scores).

Other health conditions (0 = no; 1 = yes): depression and falls

Global deterioration scale (GDS) score: ranging from very severe problem (coded as 1) to none (coded as 7). Higher score meant better cognitive functioning.

ADL limitations score: ranging from 0 to 100 (full score). The measurement tool was Modified Barthel Index. Higher score meant better functional status.

IADL limitations score: ranging from 0 to 14 (full score). The measurement tool was the Instrumental Dependency-OARS. Higher score meant better instrumental functional status.

BPSD frequency score (equal to the total frequency score of 38 symptoms): Frequency score of each symptom ranged from 0 (no occurrence) to 6 (several times an hour). The measurement tool was the Adapted Cohen-Mansfield Agitation Inventory-Community Form. Higher score meant more frequent BPSD.

### Carer variable

Carer stress score (equal to the total problem score): Each BPSD caused different levels of problems to carers, ranging from no problem (scored 0) to large problem (scored 4). Higher score meant higher care stress level.

### Data analysis

We used PASW 19.0 to perform all analyses. Descriptive data were presented using mean, minimum and maximum figures, standard deviation and proportions. We performed Cox proportional hazards regression analyses (backward step-wise) for six, nine and 16 months’ study periods respectively to examine the short-term and long-term effects of baseline variables on time to death or hospital admission.

The Cox regression analyses included two steps. Step one was univariate analysis, aiming to identify potential significant factors for time to death or hospital admission of each study period (p-value set at 0.10). Step two was multivariate analysis involving running a Cox regression model for each study period by including all of its potential significant factors. This step determined the final significant factors for time to death or hospital admission for each study period (p-value set at 0.05).

Kaplan-Meier survival curve using censored data was displayed.

Results of the Cox regression analyses were shown in the form of hazard ratios. A hazard ratio of greater than 1 for a variable indicates that hazard increases as the value of the variable increases at any period of time, and vice versa for a hazard ratio of less than 1. The 95% confidence interval, and two-tailed P-value of 0.05 were adopted.

### Sample size

The sample size for this survival analysis was estimated based on 5% statistical significance, 80% power and minimum difference to be detected of 20% between categories in subgroup independent factors. It was unclear however at study outset what number of death and hospital admission events would occur in this population in the six, nine and 16-month study periods, as well as the size of the independent factor subgroups, all these parameters being necessary for sample size estimation.

Plausible estimates of numbers of these parameters indicated that sample size estimates were sensitive to variation of numbers used and whether the study would be adequately powered. This being so, death and hospital admission events were combined to increase the number of what became the principal outcome variable. Such combination is commonly performed in other fields of health care [36]. Based upon the following plausible numbers — 15% death or hospital admissions, 70%/30% relative size of subgroups and 20% difference in subgroup effects, it was estimated that a total sample size of 237 was necessary. Assuming 20% attrition in numbers other than death and hospital admissions, the total sample size as required is 284.

Using the same method in sample size calculation but based on 10% death, 70%/30% relative size of subgroups and 10% difference, the total sample size of 433 (larger than the sample size of this study — 284) was necessary to examine risk factors for time to death. This further supports why we needed to combine death and hospital admission and primarily examined risk factors for time to death or hospital admission.

## Results

### Characteristics of the study population

#### Socio-demographic characteristics

Table 1 demonstrates that of the 284 EACHD clients, all were non-indigenous people and the majority were 65 years and older (93.3%), female (64.4%), pension recipients (85%), and born in Australia (61.6%). Approximately 30% of the clients lived alone, and over 85% spoke English as the first language at home. In addition, about 90% of the clients had co-resident or non-resident carers, and about 85% had carers who were either their partner/spouse or son/daughter-in-law.

**Table 1:** Demographics of the study sample (n = 284)

**Variables**	**n (%)**
Age	
58-64	19 (6.7)
65-74	38 (13.4)
75-84	110(38.7)
85 and over	117 (41.2)
Female gender	183 (64.4)
Non-indigenous people	284 (100.0)
Government pension (0 = private income; 1 = pension)	236 (83.1)
Living alone (living with family/others = 0; lives alone = 1 )	84 (29.6)
Carer relationships	
Relatives/friends	50 (17.6)
Partner/spouse	121 (42.6)
Son/daughter/in-law	113 (39.8)
Carer status	
No carer	30 (10.6)
Co-resident carer	185 (65.1)
Non-resident carer	69 (24.3)
Australian-born (Other = 0; Australia = 1)	175 (61.6)
Speaking English (Other = 0; English = 1)	242 (85.2)

Note: The 284 clients were aged between 58 and 100, with 19 clients aged between 58 and 64 (strictly speaking not “older people”).

#### Service use and medical conditions

According to Table 2, about 65% of the 284 clients had had GP visits, 2.1% had had ED visits, about 30% had used specialist dementia care and inpatient hospital care respectively, over 10% had used outpatient services and home nursing care respectively, and approximately 80% had used some types of community care, such as personal care, domestic care, and allied health services three months prior to the baseline survey. On average, the clients had used 1.8 hours of case management services, such as needs assessment, care planning and care coordination.

**Table 2:** Past three months’ service use and medical conditions measured at baseline

**Variables**	**n (%)**	**Mean; range**	**SD**
Prior GP visits	183 (64.4)		
Prior outpatient visits	33 (11.6)		
Prior ED visits	6 (2.1)		
Prior inpatient hospital use	87 (30.6)		
Prior home nursing use	35 (12.3)		
Prior dementia specialist care use	80 (28.2)		
Prior community care use	226 (79.6)		
**Charlson index of co-morbidity**			
No	43 (12.1)		
Mild	198 (55.9)		
Moderate	96 (27.1)		
Severe	17 (4.8)		
Depression	37 (13.0)		
Falls	25 (8.8)		
Use of case management (hours)		1.8 (0–30)	3.0
ADL score		58.6 (0–100)	24.9
IADL score		3.5 (0–9)	2.3
GDS score		3.0 (1–7)	1.0
BPSD score		27.2 (0–107)	20.7
Carer stress score		10.4 (0–55)	9.8

These clients’ physical and cognitive functioning (measured by ADL, IADL and GDS scores), and BPSD (measured by BPSD frequency score), as well as carers’ stress level (measured by carer stress score) varied substantially.

Regarding medical conditions, over 50% of clients had mild medical conditions, over 30% had moderate or severe medical conditions, 13% had depression, and approximately 10% had history of falls.

### Numbers of clients died or were admitted to hospital during different study periods

Through our analysis, of the 284 EACHD clients, during six months, 25 died, eight moved to hospital, and one moved to nursing home and died; during nine months, 29 died, nine moved to hospital, and one moved to nursing home and died; and during 16 months, 30 died, nine moved to hospital, one moved to nursing home and died, and one moved to hospital and died.

### Potential risk factors of time to death or hospital admission

Using univariate Cox regression analyses, four potential significant factors of death or hospital admission during the six months post commencement of services were GDS score (p = 0.002, HR = 0.58), ADL score (p = 0.003, HR = 0.98), inpatient hospital care use (p = 0.001, HR = 3.29), and community care use (p = 0.055, HR = 0.50).

Four potential significant factors of death or hospital admission during the nine months were GDS score (p = 0.000, HR = 0.56), ADL score (p = 0.000, HR = 0.98), IADL score (p = 0.071, HR = 0.87) and inpatient hospital care use (p = 0.001, HR = 2.93).

Seven potential significant factors of death or hospital admission during the 16 months were GDS score (p = 0.000, HR = 0.54), ADL score (p = 0.000, HR = 0.98), IADL score (p = 0.044, HR = 0.86), BPSD frequency score (p = 0.082, HR = 1.01), inpatient hospital care use (p = 0.000, HR = 3.22), carer stress score (p = 0.053, HR = 1.03), and carer relationships (p = 0.047, HR = 1.58).

### Significant risk factors for time to death or hospital admission within different study periods

Using multivariate Cox regression analyses, significant risk factors for time to death or hospital admission of six, nine and 16-month study periods were determined:

Significant factors for six-month study period included previous hospital admissions (Wald = 10.55; HR = 3.12; 95% CI of HR: 1.57-6.19; p = 0.001), GDS score (Wald = 9.12; HR = 0.58; 95% CI of HR: 0.40-0.82; p = 0.003), and community care use (Wald = 5.48; HR = 0.42; 95% CI of HR: 0.21-0.87; p = 0.019). According to their hazard ratios, having previous hospital admissions, previously worse cognitive status, and no previous use of community care increased the likelihood of clients’ early death or hospital admission.

Significant factors for nine-month study period included previous hospital admissions (Wald = 10.09; HR = 2.80; 95% CI of HR: 1.48-5.26; p = 0.001) and GDS score (Wald = 11.06; HR = 0.58; 95% CI of HR: 0.42-0.80; p = 0.001). According to their hazard ratios, having previous hospital admissions and previously worse cognitive status increased the likelihood of clients’ early death or hospital admission.

As shown in Table 3, significant factors for 16-month study period included previous hospital admissions (Wald = 10.92; HR = 2.93; 95% CI of HR: 1.55-5.53; p = 0.001) and GDS score (Wald = 4.84; HR = 0.65; 95% CI of HR: 0.45-0.96; p = 0.028). According to their hazard ratios, having previous hospital admissions and previously worse cognitive status increased the likelihood of clients’ early death or hospital admission.

**Table 3:** Significant risk factors for time to death or hospital admission during 16-month study period

**Variables during three months before commencement**	**Wald**	**HR**	**95% CI of HR**	**p**
Hospital admissions	10.92	2.93	1.55–5.53	0.001
GDS score	4.84	0.65	0.45–0.96	0.028
ADL score	2.79	0.99	0.98–1.00	0.095
IADL score	1.96	1.16	0.94–1.44	0.162
Carer stress score	2.33	1.02	0.99–1.05	0.127
Carer relationships				
Relatives/friends				
Partner/spouse	0.24	0.75	0.23–2.45	0.628
Son/daughter/in-law	0.04	1.13	0.35–3.60	0.838
BPSD frequency score	0.00	1.00	0.97–1.02	0.992

Notes:

1. All variables were measured at baseline; commencement means being enrolled and commencing using services provided by EACHD program.

2. Having hospital admissions during the past three months = 1; having no admissions = 0. GDS score: 1–7; higher score, better cognitive status. ADL score: 0–100 (full score); higher score, better functional status. IADL score: 0–14 (full score); higher score, better instrumental functional status. BPSD frequency score: 0–228 (full score: 6*38); higher score, more frequent BPSD. Carer stress score: 0–152 (full score: 4*38); higher score, higher care stress level.

3. HR (hazard ratio) > 1 indicates increased risk of the occurrence of an event; HR < 1 means decreased risk of the occurrence of an event.

4. -2 Log Likelihoods in the final model and null model were respectively 374.235 and 379.595.

We were particularly interested in the effects of the severity of clients’ medical conditions on death or hospital admission. Univariate analyses indicated that this variable was not a potential significant factor for each study period (p = 0.389 for six-month, p = 0.118 for nine-month, and p = 0.142 for 16-month). But we performed additional multivariate analyses by including this variable together with other potential significant factors for each study period. Again, these analyses showed that this variable was not a significant factor of death or hospital admission of each study period.

### Potential and significant risk factors for time to death within different study periods

During six-month study period, potential significant factors included previous GDS score (p = 0.011; HR = 0.6), ADL score (p = 0.001; HR = 0.98), hospital admissions (p = 0.012; HR = 2.67), and community care use (p = 0.05; HR = 0.45). Main results of the multivariate analysis included: previous hospital admissions (Wald = 4.32; HR = 2.29; 95% CI of HR: 1.05-4.98; p = 0.038) and ADL score (Wald = 9.00; HR = 0.98; 95% CI of HR: 0.96-0.99; p = 0.003).

During nine-month study period, potential significant factors included previous GDS score (p = 0.007; HR = 0.61), ADL score (p = 0.000; HR = 0.98), IADL score (p = 0.041; HR = 0.83), hospital admissions (p = 0.026; HR = 2.26), carer stress score (p = 0.038; HR = 1.03), and carer relationships (p = 0.042; HR = 1.77). Main results of the multivariate analysis included: previous hospital admissions (Wald = 3.36; HR = 1.99; 95% CI of HR: 0.95-4.13; p = 0.067) and previous ADL score (Wald = 12.04; HR = 0.98; 95% CI of HR: 0.96-0.99; p = 0.001).

During 16-month study period, potential significant factors included previous GDS score (p = 0.003; HR = 0.58), ADL score (p = 0.000; HR = 0.98), IADL score (p = 0.038; HR = 0.83), hospital admissions (p = 0.014; HR = 2.43), carer stress score (p = 0.039; HR = 1.03), and carer relationships (p = 0.026; HR = 1.86). Main results of the multivariate analysis included: previous hospital admissions (Wald = 4.14; HR = 2.12; 95% CI of HR: 1.03-4.36; p = 0.042) and ADL score (Wald = 12.31; HR = 0.98; 95% CI of HR: 0.96-0.99; p = 0.000).

To sum up, the multivariate analyses indicated that having previous hospital admissions increased the likelihood of clients’ early death during the six and 16 months’ study periods. Previously worse physical functioning (lower ADL score) was not a significant risk factor as its hazard ratio during each study period was very close to 1:00.

### Kaplan-Meier analysis

**Figure 1:**
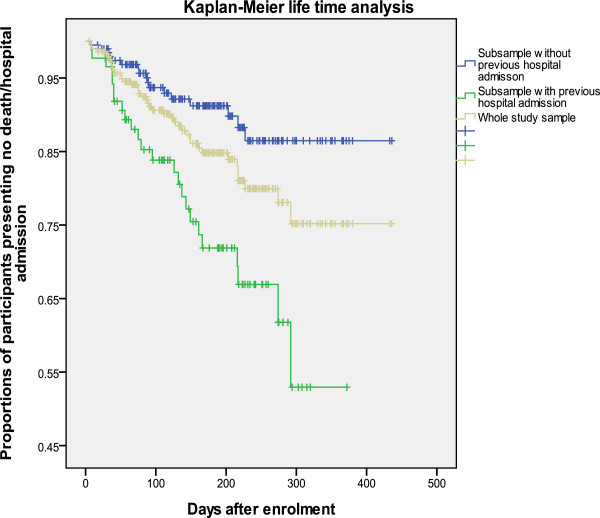
Kaplan-Meier survival curves of the study sample and sub-samples by having previous hospital admissions or not.

**Figure 2:**
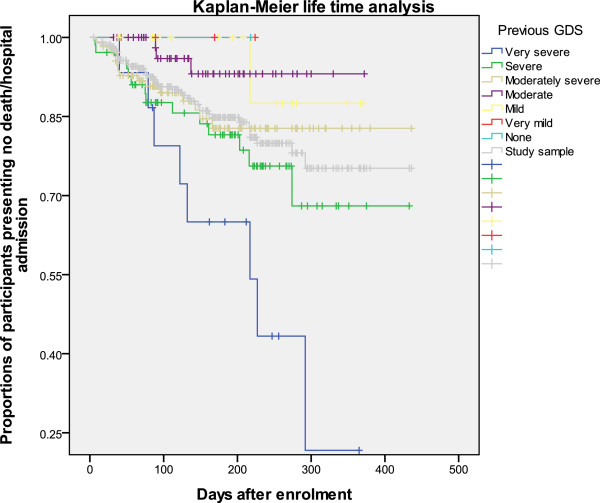
Kaplan-Meier survival curves of the study sample and sub-samples by previous GDS.

**Figure 3:**
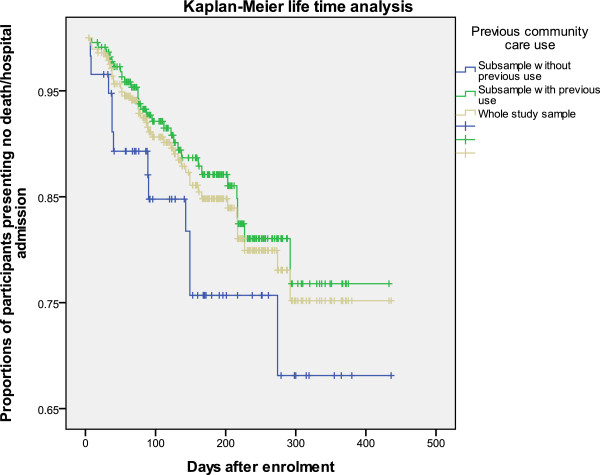
Kaplan-Meier survival curves of the study sample and sub-samples by having previous use of community care services or not.

Figures 1, 2 and 3 show the patterns of the whole study sample and sub-samples presenting death or hospital admission during the 16-month study period. Figures 1 and 2 support the results described above that previous (three months prior to using EACHD program services) hospital admissions and previously worse cognitive status increased the likelihood of earlier death or hospital admission. Figure 3 supports that previous use of community care did not reduce the likelihood of early death or hospital admission during the nine- and 16-month study periods.

## Discussion

To our best knowledge, this study is one of few longitudinal studies that focused on risk factors for death or hospital admission in the short term (six and nine months) and long term (16 months). In addition, it particularly targeted community-based frail older Australians living with dementia.

Across the three different study periods, hazard ratios for previous hospital admissions (6-month: HR = 3.12; 9-month: HR = 2.8; 16-month: HR = 2.9) and cognitive status (6-month and 9-month: HR = 0.58; 16-month: HR = 0.65) were quite similar. These results test the assumption of the proportional hazard model that previous hospital admissions, and previously worse cognitive status have consistent significant independent effects on time to death or hospital admission over time [37]. In this aspect, few other studies are able to test the assumption in the way we have done here.

Based on those hazard ratios, previous hospital admissions was a strong risk factor. This result supports a previous review study, reporting that prior hospital admissions are significantly associated with subsequent hospital admission. The main reason is that older people having preceding inpatient hospital admissions normally have worse health condition and thereby being more likely to be admitted to hospital later [12].

We found that previously worse cognitive status was a moderately strong risk factor. Even though we focused on different outcome measure (death or hospital admission) and special population (community-dwelling older people living with dementia), our finding can provide some evidence for previous review and research studies, indicating that previously worse cognitive status is a significant risk factor of such adverse health events as the permanent nursing home admission and death among frail older people [1,9,10,16].

No previous use of community care was identified as a moderate risk factor (HR = 0.42) for death or hospital admission within six months’ study period in our study. This suggests that preceding use of community care has a protective effect on clients’ subsequent death or hospital admission in the short term but not in the long term. This is an important finding because it provides evidence for the importance of providing community care services for this vulnerable client population living with dementia. The literature has agreed that use, more use, and more frequent use of some types of community care services can significantly reduce the risk of death later [18].

Our study did not indicate significant effects of the severity of clients’ medical conditions, depression and falls on time to death or hospital admission within any study periods. To this end, a review study has reported that depression is a consistent predictor of nursing home admission for people living with dementia [8]. Another study has found that both heart disease and cancer (coded as 1 = “yes” and 0 = “no”) are predictors of death for people aged 70 and over [15]. A third study has reported that having one or more medical conditions (Charlson index includes no, one, and over one medical conditions) reduces the risk, but having depressive disorder increases the risk of nursing home admission [11]. It is not surprising that these studies report mixed results because they focus on different outcome variables, predictor variables, and/or client groups. More research with clarified research designs in these aspects is needed to explore the impact of medical conditions on clients’ adverse health events in the future.

Our findings regarding having previous hospital admissions as a strong risk factor for time to death during six-month (HR = 2.29) and 16-month study periods (HR = 2.12) further confirm the importance of focusing on this risk factor in the management of frail older people living with dementia.

Previously worse cognitive functioning (lower GDS score) and worse physical functioning (lower ADL score) were not identified as risk factors for time to death across the three study periods. The literature has not reached agreement on whether functioning impairment, in particular ADL, is a risk factor for client mortality depending on what measurement tools are used [19]. Our interpretation was that the impact of previous functioning impairment (functioning factors) on a medical event (death) might be trivial compared with previous hospital admissions (a medical factor). As described above, since the sample size is not adequate enough to examine risk factors for time to death, we felt unable to make further comments.

### Significant risk factors for time to death or hospital admission over short- and long-term study periods

The literature has reported that the length of follow-up may determine when risk factors emerge [6]. Given the lack of theoretical and empirical studies exploring the optimum study period for examining risk factors, we were unable to explain why different variables did or did not emerge as significant risk factors at different times.

It should be noted that in our study most events occurred during six months. The literature has reported that EACHD clients typically use the EACHD program services for six months or even a shorter time as their health condition deteriorates fast; therefore they access other care services (e.g. moving to nursing homes) that can better meet their increased care needs [26].

Based on the literature cited above and our findings, it is not surprising that the risk values of previously worse cognitive status and previous hospital admissions do not change over time. Regarding why no previous use of community care services appearing as a short-term risk factor, our interpretations included:

First, the use of community care alone may not achieve long-term protective effect for older people living with dementia. This is because the illness of older people living with dementia progresses fast; therefore they need evolving and even special care services that take into account of the change of their physical and cognitive functioning, behaviour, emotion, and dementia related illness [38].

Second, the amount, frequency and time of the use (rather than use or not) of community care services may have more impact on the duration of the protective effect. However, this study could not test this assumption as the original project had not collected information on clients’ use of care services in such detail. Future research is needed to clarify this issue.

In any event, both theoretical and empirical studies are warranted to explore when older people living with dementia face what risk factors, so care professionals can implement target interventions at the right time to decelerate their progressive deterioration and associated early adverse events such as death or hospitalization [39].

A related analysis would be to analyse the effects of time-dependent variables (ADLs, IADLs, GDS, BPSD and carer stress involved in our study) on death or hospital admission because previous studies identify that such time-dependent variables as dementia, cognitive status measured by MMSE (Mini–mental state examination), ADL and IADL impairment, social support, and number of prescription medications are predictors of nursing home placement [13,14,20]. We did not conduct this analysis because it was peripheral to the research question addressed here and the complexity of the data set presented some technical difficulties for that approach.

### Limitations

This study has some limitations. As a longitudinal study, the overall study period (16 months) would ideally have been longer. This may have impact on the findings although the consistency of our findings across the time points studied strengthened the conclusions. The literature has indicated that studies with a longer study period seem more likely to find significant results though this conclusion is yet to be proved [8].

Due to third party recruitment, it was not possible to determine a precise response rate for the sample size; hence the enrolled 354 EACHD clients of the original project may not represent the overall community-dwelling older people living with dementia. But a preliminary comparison between this study sample and that of a national data set indicated that this sample was comparable on basic available demographic variables [40].

It is noteworthy that the original research project did not involve indigenous people. Research targeting this population is needed because there is a higher prevalence of dementia among indigenous Australians [41], current policies emphasize providing more aged care places for indigenous Australians [42], and indigenous people living with dementia have different health conditions and care needs, and face different risk factors [43,44].

Relying on available information, we were unable to examine some variables which may have potential impact on clients’ adverse health events. These include clients’ treatment status, carers’ characteristics and well-being, case managers’ characteristics, agency and system factors, and policy resources [12,17,20,45-47].

Ideally, death and hospital admission should be treated as two independent outcome variables. We combined them as one endpoint because the numbers of the two events were very small within any study periods. In the original project, clients would be discharged from EACHD program when they could not continue to use the program services due to death, or unavoidable permanent nursing home or hospital admission [26]. From this perspective, the permanent nursing home admission and the worse event—hospital admission—may be closely related to death at least in our study sample. In addition, as described previously, to examine risk factors for time to this combined outcome measure, the sample size of our study (n = 284) was sufficient to detect effects of 20% differences (though not smaller differences) in categories of independent variables tested.

As a matter of fact, some studies report that the permanent nursing home admission is a significant risk factor of death for people living with dementia or Alzheimer’s disease [13,14]. Hence, combining death and hospital admission in our study may not have had a very significant impact on the results. However, future research should examine risk factors of death and hospital admission separately if possible.

### Implications

This study has some implications for case managers’ practice. According to our findings, case managers should continuously focus on one short-term protective factor (previous use of community care services) and two consistent risk factors (preceding hospital admissions and previously worse cognitive status) when enrolling clients. This will delineate individuals who are at high risk of subsequent death or hospital admission and thereby needing special attention for targeted interventions.

## Conclusions

For community-dwelling older people living with dementia, having previous hospital admissions was a consistent risk factor for time to death or hospital admission, and for time to death respectively. Previously worse cognitive status was a consistent risk factor for time to death or hospital admission. Both risk factors need target interventions by case managers. Meanwhile, having previous use of community care as a short-term protective factor for time to death or hospital admission is noteworthy.

## Competing interests

The authors declare they have no competing interests.

## Authors’ contributions

EY, DD and CD conceived the analysis presented here. EY drafted the manuscript, and DD and CD were involved in analysing and interpreting the data as well as revising the manuscript. CD and VW designed the original project, and undertook data collection. SVH was involved in the design of statistical methods. All authors read and gave final approval for this manuscript to be published and agreed to be accountable for all aspects of the work.

## Pre-publication history

The pre-publication history for this paper can be accessed here:

http://www.biomedcentral.com/1471-2318/14/71/prepub
